# Gastrodin attenuates perfluorooctanoic acid-induced liver injury by regulating gut microbiota composition in mice

**DOI:** 10.1080/21655979.2021.2009966

**Published:** 2021-12-04

**Authors:** Shumin Ma, Yanyan Sun, Xueting Zheng, Yang Yang

**Affiliations:** a Department of Hepatology, Shandong Provincial Public Health Clinical Center, Jinan, China; b Fever Observation Ward, Shandong Provincial Public Health Clinical Center, Jinan, China; c Department of Gastroenterology, People’s Hospital of Yangxin County, Binzhou, China

**Keywords:** Perfluorooctanoic acid, liver injury, gastrodin, gut microbiota, short-chain fatty acids

## Abstract

Perfluorooctanoic acid (PFOA) can accumulate in the livers of humans and animals via the food chain, resulting into liver injury, which is closely related to intestinal flora dysbiosis. Gastrodin has been reported to have hepatoprotective effect. However, whether gastrodin can alleviate PFOA-induced liver injury via modulating gut microbiota remains unclear. Herein, a PFOA-induced liver injury model was established by gavage of PFOA (5 mg/kg body weight) in 2% Tween 80 solution once daily for 6 weeks in mice, and then gastrodin in saline (20 mg/kg body weight) was used once daily for 8 weeks to treat liver damage. The biochemical indexes associated with liver function, oxidative stress, and inflammatory factors were examined. Hematoxylin-eosin staining was used to determine the liver histopathological changes. Besides, 16S rRNA sequencing was used to analyze the difference of gut microbiota between the model and treatment groups. The results showed that gastrodin significantly improved the oxidative stress caused by PFOA. Intestinal flora analysis showed that gastrodin treatment significantly increased the relative abundance of probiotics, such as *Lactobacillus, Bifidobacterium,* and *Bacteroides*, while the harmful bacteria, including *Desulfovibrio* were decreased. Gastrodin treatment also significantly increased the level of short-chain fatty acids (SCFAs), such as butyric acid and isobutyric acid. Spearman correlation analysis showed that the composition changes of gut microbiota and SCFAs increase were both beneficial to alleviate the liver injury caused by PFOA. To sum up, gastrodin can effectively alleviate PFOA-induced liver injury through regulating gut microbiota composition.

## 1. Introduction

Perfluorooctanoic acid (PFOA) is widely used in industrial production [1]. owing to the wide application and extremely strong stability, PFOA is frequently detected in various environments [2]. PFOA is easily absorbed by humans and animals via the food chain and then accumulates in the liver, causing liver damage [3–6]. In a recent study, liver injury caused by PFOA exposure in mice is closely related to intestinal flora dysbiosis [7]. Gut microbiota dysbiosis can destroy the integrity of intestinal barrier and lead to the displacement of flora and leakage of intestinal derivatives, thereby accelerating the development of liver diseases through the intestinal-liver axis [8,9]. An increasing number of studies have shown that abundance decrease of beneficial intestinal bacteria, such as *Lactobacillus* and *Bifidobacterium sp*. and abundance increase of conditional pathogenic bacteria including *Enterococcus* can aggravate the progression of liver diseases [10–12]. Therefore, regulating gut microbiota composition may be an effective strategy to prevent and treat liver diseases [13].

Gastrodin is a water-soluble organic compound isolated from the root of *Gastrodia elata Blume*, and anti-inflammatory, anti-oxidation, anti-virus, and anti-tumor effects of gastrodin have been widely confirmed in experimental animal models [14–16]. Gastrodin can also alleviate hepatic fibrosis caused by bile duct ligation via improving oxidative stress and inflammatory reactions [17–19]. More importantly, gastrodin has also been shown to inhibit the inflammatory responses in mice with early atherosclerosis by remodeling intestinal microflora [20]. However, whether and how gastrodin exerts protective effects on PFOA-induced liver injury via modulating gut microbiota remains unclear.

Based on the above results, gastrodin is speculated to exert protective effects on PFOA-induced liver injury via modulating gut microbiota. To validate this hypothesis, the PFOA-induced liver injury model was established by gavage of PFOA in C57BL/6 J mice, and then gastrodin was used to treat liver damage. Besides, 16S rRNA sequencing was used to analyze the difference of gut microbiota between the model and treatment groups. The findings indicated that gastrodin alleviate liver injury caused by PFOA exposure via modulating gut microbiota composition. These findings will broaden our understanding of protective liver drugs exploration.

## 2. Materials and Methods

### 2.1 Experimental animals and treatment

Thirty-six male-specific pathogen-free (SPF) C57BL/6 J mice (6 weeks old, 18–22 g) were purchased from the Animal Experimental Center of Shandong University (Jinan, China). Mice were raised in a sterile environment (temperature: 22 ± 3°C; humidity: 55 ± 5%; 12-hour light/dark alternation), and had free access to standard feed (Charles River, Beijing, China) and water. All animal experiments performed in this study were approved and supervised by the Ethics Committee of Shandong Provincial Public Health Clinical Center (Jinan, China).

After 1 week of adaptation, 36 mice were randomly divided into 3 groups with 4 replicates per group and 3 mice per replicate. As previously described [7], the experimental design and model establishment are shown in Figure S1. (1) Control group (NC): 0.5 ml of 2% Tween 80 solution was given by gavage once a day for 6 weeks, and then 0.5 ml of sterile saline was given once daily for 8 weeks; (2) Model group (PT): mice administered 5 mg PFOA/kg (Sigma-Aldrich, Darmstadt, Germany) body weight in 2% Tween 80 solution (0.5 ml) by orally gavage once daily for 6 weeks, and then 0.5 ml sterile saline was given by gavage once a day for 8 weeks. (3) Gastrodin treatment group (PG): mice administered 5 mg PFOA/kg body weight in 2% Tween 80 solution (0.5 ml) by gavage once daily for 6 weeks, and then gastrodin at 20 mg/kg body weight (Tianjin Shilan Technology Co., Ltd., Tianjin, China) in sterile saline (0.5 ml) was given once daily for 8 weeks [18]. When the experiment was completed, after 12 h of fasting, mice were intraperitoneally injected with 1% pentobarbital sodium solution, followed by cervical dislocation. Blood samples were collected and stored at room temperature for 30 min, followed by centrifugation at 3 000 × g for 15 min. Then, sera were collected and stored at −80°C. Mice were dissected, and the body and liver weight were recorded. The cecum contents were collected and stored at −80°C, and liver lobes harvested were placed into 10% formalin solution for histopathological analysis. Other liver tissues were immediately frozen in liquid nitrogen and stored at −80°C.

### 2.2 Histopathological analysis

Liver samples fixed in formalin were dehydrated, embedded in paraffin, and cut into of 5-μm-thick sections. Sections were stained with hematoxylin and eosin (H&E) and imaged by an Olympus ix73 optical microscope (Olympus, Tokyo, Japan). Image-Pro Plus software 6.0 (Media Cybernetics, Rockville, MD, USA) was used to select 5 random fields of views for each slice to calculate the ratios of pathological areas (vacuolation, inflammation, and necrosis) vs. total areas [21]. As previously described [22], the histological activity index (HAI) of liver tissue was analyzed to evaluate the degree of liver injury.

### 2.3 Determination of serum transaminase

The serum levels of aspartate amino transferase (AST) and alanine amino transferase (ALT) were analyzed by a Hitachi 7600 type automated analyzer (Hitachi High-technologies corporation, Tokyo, Japan).

### 2.4 Determination of oxidative stress in the liver

Liver tissue samples were homogenized in precooled 0.85% normal saline by 9 times the volume to obtain a 10% (w/v) liver homogenate. Then, the homogenate was centrifuged at 3 000 × *g* at 4°C for 10 min to obtain supernatant for further analysis. The commercial kits (Nanjing Jiancheng Biotechnology Co., Ltd (Nanjing, China), were used to determine the activities of superoxide dismutase (SOD), glutathione peroxidase (GSH-Px) and catalase (CAT), and the level of malonyldialdehyde (MDA) in liver homogenates. Protein concentrations were determined by the BCA protein assay kit (Solarbio life science, Beijing, China). The level of each index was calculated based on the calculation formula provided with the kit.

### 2.5 Examination of serum inflammatory factors

Enzyme-linked immunosorbent assays (ELISA) kits (Nanjing SenBeiJia Biological Technology co., Ltd., Nanjing, China) were employed to determine the contents of tumor necrosis factor-alpha (TNF-a) and interferon-beta (IFN-β) in serum samples.

### 2.6 SCFAs content analysis

Analysis of SCFA content was conducted based on high performance liquid chromatography method [23]. In brief, a total of 20 mg cecal content was homogenized with 1 ml deionized water, centrifuged at 14,000 × *g* for 10 min, and the supernatant was collected and filtered through a 0.22-μm filter. Then, 10% (v/v) 0.01 mol/L sulfuric acid was added for acidification. The concentration of SCFAs (acetic acid, propionic acid, butyric acid, isobutyric acid, valeric acid, and isovaleric acid) was determined by a Waters Alliance e2659 high performance liquid chromatography (Waters, Milford, MA, USA) equipped with a Waters XBridge C18 reversed-phase column (5 µm, 4.6 × 250 mm) and a Waters 2489 UV/Vis detector. The mobile phase was sulfuric acid (0.01 mol/L), the flow rate was 1.0 ml/min and the column temperature was 30°C. The SCFAs were identified by comparing the peak residence time of samples with the standard, and the concentration was determined by the external standard calibration method of Waters Empower 3. The SCFA concentration was expressed as μg/g sample.

### 2.7 Intestinal microbial analysis

DNA of cecal content was extracted by QIAamp Fast DNA Stool Mini Kit (Qiagen, CA, USA). The V3-V4 region of 16S rDNA gene was amplified with primers 341 F (5ʹ-CCTAYGGGRBGCASCAG-3ʹ) and 806 R (5ʹ-GGACTACNNGGGTATCTAAT-3ʹ), and the PCR products were purified with a QIAquick Gel Extraction Kit (Qiagen, CA, USA) and sequenced using an Illumina MiSeq platform (Illumina, CA, USA).

The 16S rDNA sequencing data were analyzed by QIIME Conduct analysis [24]. In brief, Trimmomatic v0.39 [25] was adopted to perform the quality control of raw data, and FLASH v1.2.11 [26] was employed to merge the clean reads after quality control (minimum overlap of 10 bp). Based on 97% sequence similarity, operational tax units (OUT) clustering was conducted by Vsearch v2.15.0 [27]. OUT after clustering was classified and annotated through comparison with the Silva database [28] (Release 138) using RDP Classifier v2.2 [29]. The alpha diversity indexes Shannon, Simpson and Chao1 of intestinal microbes were calculated by Mothur (https://mothur.org/). Furthermore, Principal Coordinates Analysis (PCOA) based on Bray-Curtis dissimilarity [30] was used to compare the differences in beta diversity of bacterial communities in different samples.

### 2.8 Statistical analysis

The data were analyzed by SPSS 22.0 software (SPSS Inc., IL, USA). Firstly, the Kolmogorov–Smirnov test was used to determine the normality of the data distribution. Then, the Kruskal–Wallis test (for non-normal distribution) or one-way ANOVA (for normal distribution) was adopted to evaluate the differences between groups. The Spearman’s rank correlation was utilized to analyze the correlation between the indicators of liver injury and changes of intestinal flora composition and SCFA level. Data were expressed as the mean ± standard deviation (M± SD). *P* ≤ 0.05 was considered statistically significant.

## 3. Results

### 3.1 Gastrodin relieves PFOA-induced liver injury in mice

**Figure 1.**
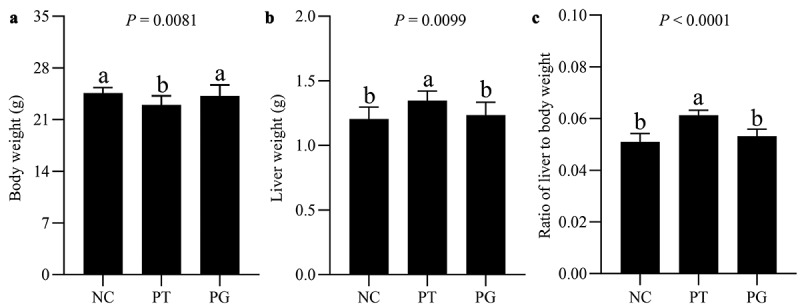
Gastrodin ameliorated PFOA-induced hepatomegaly. (a) Body weight, (b) liver weight and (c) the ratio of liver to body weight. NC group, normal control mice treated with vehicle; PT group, PFOA-induced liver injury mice treated with normal saline; PG group, PFOA induced liver-damaged mice were treated with gastrodin for 8 weeks. Values are represented as mean ± standard deviation (M± SD). Different superscript letters within a column showed significant difference (*P* < 0.05)

To analyze the therapeutic effect of gastrodin on PFOA-induced liver injury, we established a mouse model of PFOA-induced liver injury and studied the effect of gastrodin on PFOA-induced liver injury (Figure S1). Compared with the NC, mice exposed to PFOA had lighter body weight (Figure 1a) and enlarged livers (Figure 1b), and the ratio of liver to body weight was significantly increased (Figure 1c). However, gastrodin treatment significantly reduced the ratio of liver to body weight, and alleviated the hepatomegaly caused by PFOA exposure.

**Figure 2.**
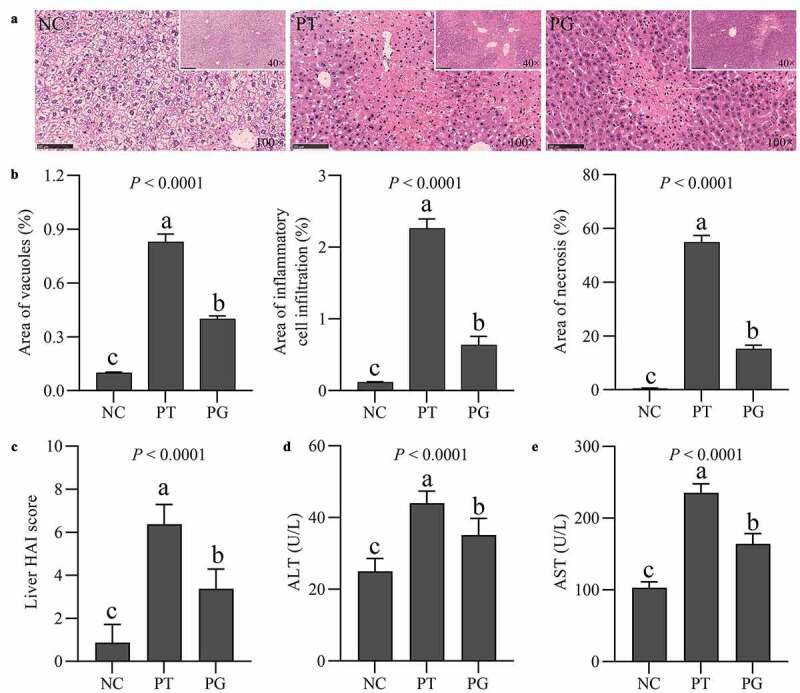
Gastrodin improved PFOA-induced liver injury. (a) Histopathological changes of the liver stained with H&E. (b) The vacuole, infiltration and necrotic areas and (c) HAI score in H&E stained sections. Serum levels of (d) ALT and (e) AST. Values are represented as the mean ± standard deviation (M± SD). Different superscript letters within a column showed significant difference (*P* < 0.05)

As shown in Figure 2a, mice in the NC group, the liver presented normal structure with well-preserved cell morphology and prominent nucleus. After PFOA exposure, the liver of mice in the PT group was obviously damaged, showing vacuolation, regional inflammatory cell infiltration, and necrosis. However, after gastrodin treatment, the histopathological changes were alleviated, and the vacuolation, infiltration and necrosis areas in the liver were reduced (Figure 2b). Gastrodin treatment improved the liver histological lesions induced by PFOA exposure, and the HAI score of liver was significantly decreased (Figure 2c). Gastrodin also decreased the enzyme activities of AST and ALT in serum (Figure 2d, e). The above results indicated that gastrodin improves liver injury caused by PFOA exposure.

### 3.2 Gastrodin reduces oxidative stress of the mouse liver induced by PFOA

**Figure 3.**
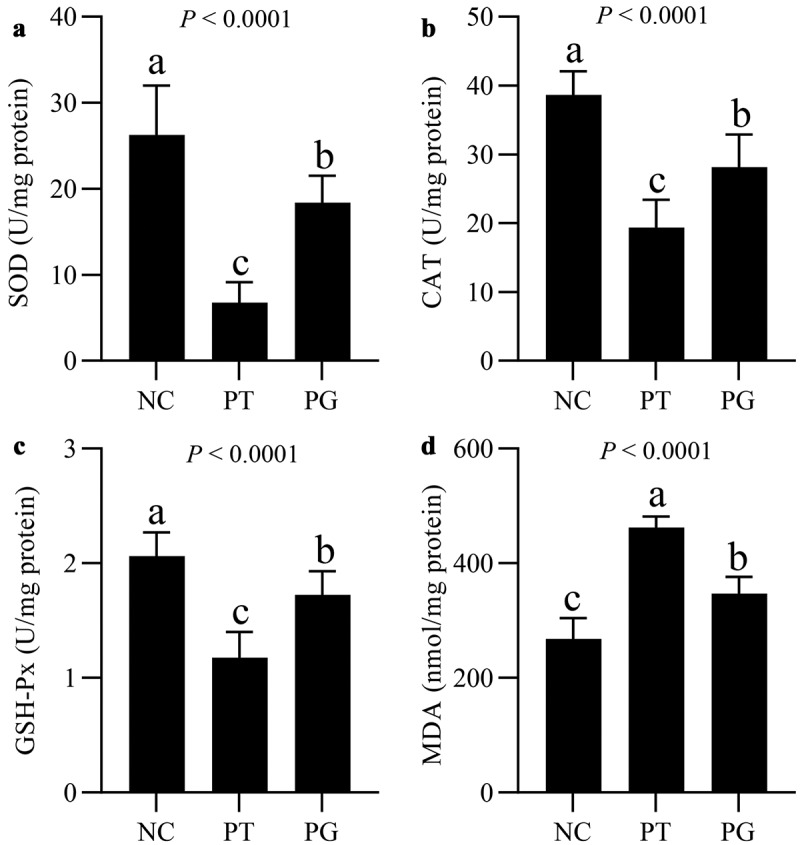
Gastrodin protected against PFOA-induced oxidative stress. Hepatic levels of (a) SOD, (b) CAT, (c) GSH-Px and (d) MDA, respectively. Values are represented as mean ± standard deviation (M± SD). Different superscript letters within a column showed significant difference (*P* < 0.05)

To explore whether the protective effect of gastrodin on the liver is related with the decrease of oxidative stress, the levels of antioxidant enzymes and MDA in liver were determined. Compared with the PFOA exposure group, the activities of SOD, CAT, and GSH-Px in mice in the PG group were significantly increased. On the contrary, compared with the exposure group, the level of MDA in the PG group was significantly decreased (Figure 3). The above results suggested that gastrodin treatment significantly inhibited the oxidative stress induced by PFOA in the liver.

### 3.3 Gastrodin restored the serum level of inflammatory cytokines

To explore whether the protective effect of gastrodin on the liver is related to the decrease of inflammatory reaction, the levels of inflammatory cytokines were determined. Compared with the NC group, the levels of TNF-α and IFN-γ in the PT group were increased significantly. After gastrodin treatment, the levels of TNF-α and IFN-γ basically returned to normal levels (Table 1). Thus, the above results indicated that gastrodin treatment alleviated the inflammatory reactions caused by PFOA exposure.

**Table 1.** Serum levels of inflammatory cytokines following exposure to PFOA with or without gastrodin administration

Inflammatory cytokines	Treatments			Statistics	
	NC	PT	PG	*SEM*	*P* value
TNF-α (ng/L)	56.27^b^	75.53^a^	60.17^b^	1.930	< 0.0001
IFN-γ (ng/L)	66.99^b^	78.44^a^	71.03^b^	1.721	< 0.0001

^a−b^In each row, the same letter means no significant difference (*P* < 0.05). SEM, Standard error of means.

### 3.4 Gastrodin enhanced SCFA level in cecal contents

To explore whether the protective effect of gastrodin on the liver is related to the SCFAs concentration, the level of SCFAs was determined. The exposure of PFOA resulted in a decrease in SCFAs in the PT group (Table 2, Figure S2). The level of butyric acid decreased most significantly, followed by isobutyric acid, valeric acid, and isovaleric acid. However, the level of SCFAs in the PG group increased significantly. The above results indicated that PFOA exposure significantly reduced the level of SCFAs, and this trend was largely reversed by gastrodin treatment.

**Table 2.** Cecum levels of short-chain fatty acids following exposure to PFOA with or without gastrodin administration

SCFA	Treatments			Statistics	
	NC	PT	PG	*SEM*	*P* value
Acetic acid (μg/g)	1418.7	1411.2	1463.7	23.332	0.630
Propionic acid (μg/g)	216.7	212.1	223.2	2.587	0.218
Butyric acid (μg/g)	624.5^a^	185.0 ^c^	421.8^b^	37.997	< 0.0001
Isobutyric acid (μg/g)	70.4^a^	39.3 ^c^	55.5^b^	2.897	< 0.0001
Valeric acid (μg/g)	124.4^a^	66.7 ^c^	96.9^b^	5.477	< 0.0001
Isovaleric acid (μg/g)	528.5^a^	385.5 ^c^	480.2^b^	15.316	< 0.0001

^a−c^In each column, means with the same letter are not significantly different (*P* < 0.05).

SEM, Standard error of means.

### 3.5 Gastrodin improved disordered intestinal flora in the PFOA mouse model

**Figure 4.**
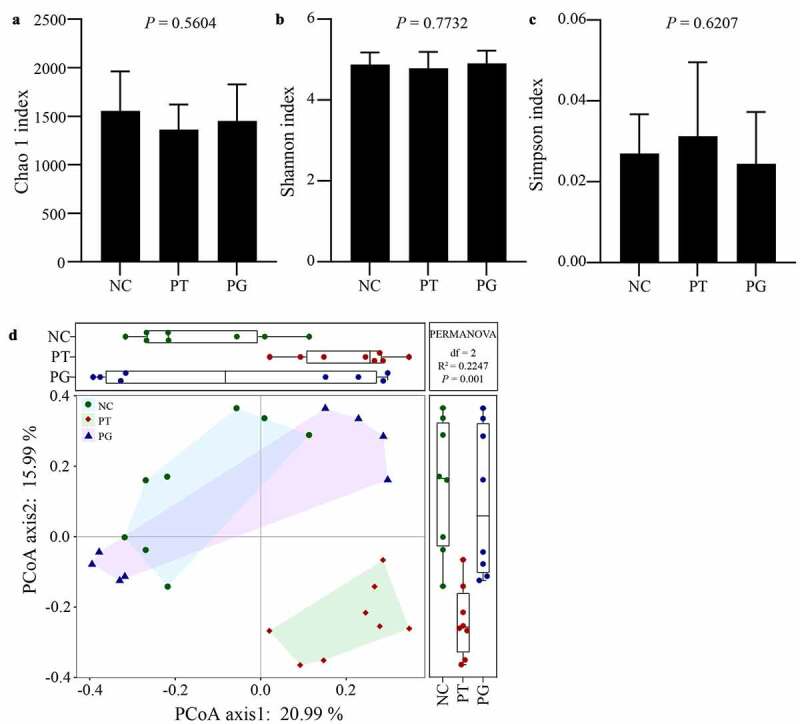
Gastrodin changed the gut microbiota diversity. Alpha diversity analysis characterized by the (a) Chao1, (b) Shannon and (c) Simpson indices, respectively. (d) Beta diversity was characterized by a Braye-Curtis analysis depending on the levels of operational taxonomic units. Values are represented as mean ± standard deviation (M± SD). Different superscript letters within a column showed significant difference (*P* < 0.05)

To explore whether the protective effect of gastrodin on the liver is related with regulating intestinal flora, 16S rRNA sequencing was used to analyze the difference of gut microbiota between the model and treatment groups. The result showed that 2,018,377 reads were obtained, and 14,437 OTUs were clustered based on a similarity of 97%. PFOA exposure and gastrodin treatment did not lead to significant differences in the diversity of gut microbiota (Figure 4a-c). PCOA results demonstrated that the composition of microbiota in the PG group was relatively closer to that in the NC group (Figure 4d).

**Figure 5.**
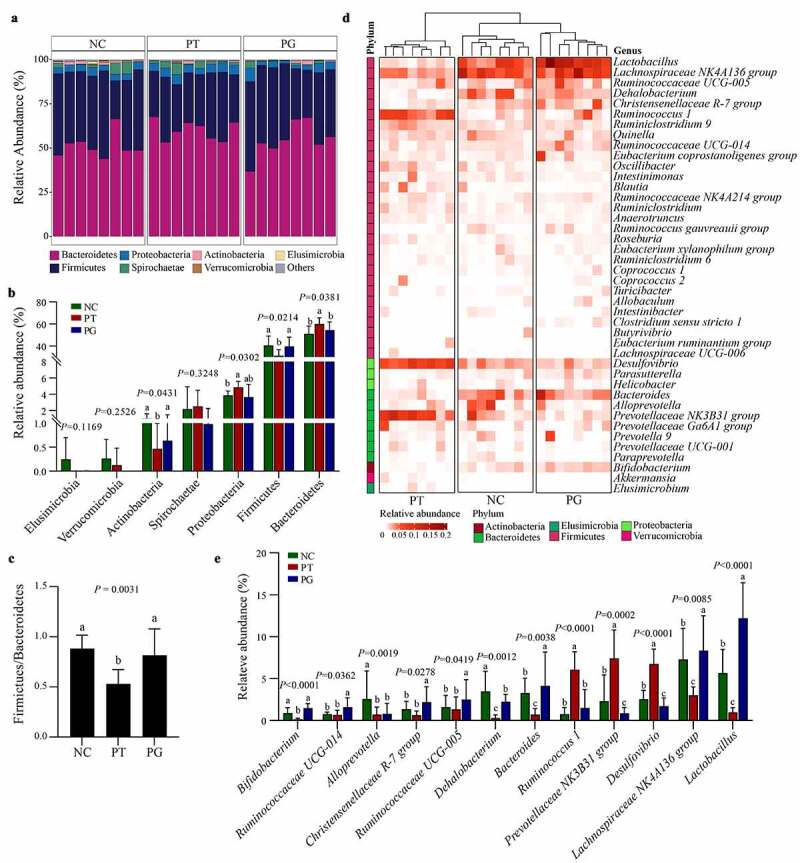
Gastrodin remodeled the composition of gut microbiota. (a) Stacked bar plot of the gut microbiota at the phylum level. (b) Changes in the abundance of specific microbes at the phylum level. (c) The ratio of Firmicutes to Bacteroidetes (F/B). (d) Heatmap of most abundant genus (relative abundance ≥ 0.01), color coded by phylum following the legend in panel. (e) Changes in the abundance of specific microbes at the genus level. Values are represented as mean ± standard deviation (M± SD). Different superscript letters within a column showed significant difference (*P* < 0.05)

At the phylum level (Figure 5a-b), the highest abundance among all groups were Bacterioides, Firmicutes, Proteobacteria, and Spirochaetae. Compared with the NC group, the relative abundance of Bacteroidetes and Proteobacteria was increased after PFOA exposure, while the relative abundance of Firmicutes and Actinobacteria was reduced. Compared with the PT group, the relative abundance of Bacteroidetes and Proteobacteria in the PG group significantly decreased, while the relative abundance of Firmicutes increased significantly. In addition, compared with the NC group, the ratio of Firmicutes/Bacteroidetes (F/B) in the PT group decreased significantly, but gastrodin can reverse the decrease of F/B ratio caused by PFOA exposure (Figure 5c).

At the genus level (Figure 5d-e), compared with the NC group, the relative abundance of *Lactobacillus, Lachnospiraceae NK4A136 group, Bacteroides, Dehalobacterium*, and *Bifidobacterium* in the PT group was obviously decreased, while the relative abundance of *Desulfovibrio, Prevotellaceae NK3B31 group*, and *Ruminococcus 1* increased significantly. After administration of gastrodin, the relative contents of *Lactobacillus, Lachnospiraceae NK4A136 group* and *Bacteroides* were significantly increased. These results showed that gastrodin can significantly improve disturbance of intestinal flora caused by PFOA exposure.

### 3.6 Correlation between indicators of liver injury and changes of intestinal flora composition and SCFAs level

**Figure 6.**
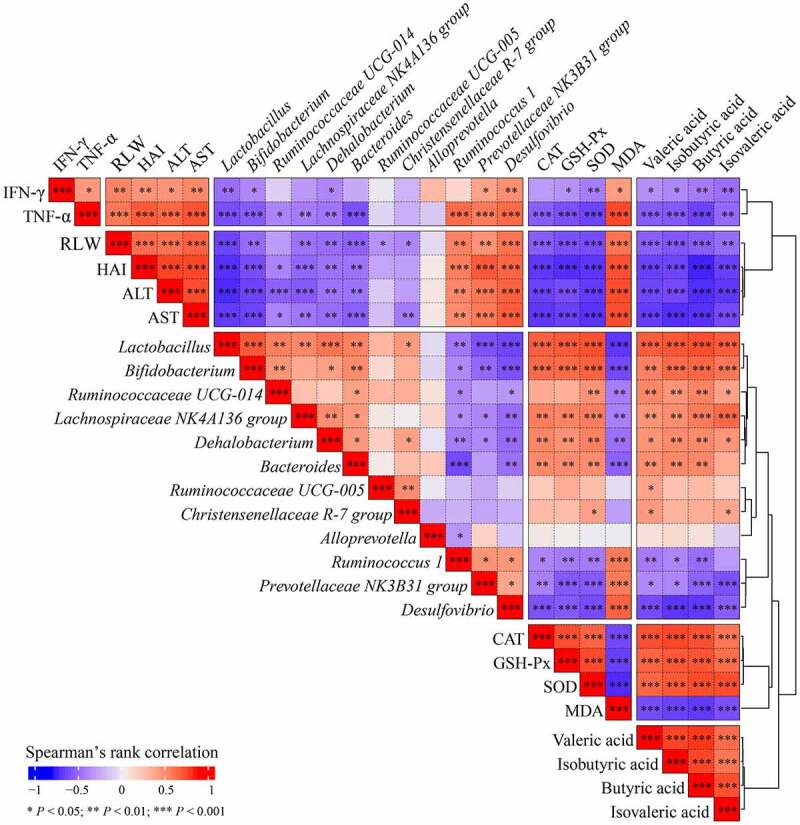
Correlation between indicators of liver injury and changes of intestinal flora composition and SCFAs level was determined using Spearman’s rank correlation analysis

To further clarify the correlation between indicators of liver injury and changes of intestinal flora composition and SCFAs level, we calculated the correlation via Spearman’s rank correlation analysis (Figure 6). The result showed that probiotics (*Lactobacillus Lachnospiraceae NK4A136 Group, Dehalobacteria, Bacterioids*, and *Bifidobacterium*) have a significant negative correlation with liver transaminase (ALT and AST; *P* < 0.001), pathological score (HAI; *P* < 0.001), the ratio of liver to body weigh (relative liver weight, RLW; *P* < 0.001), inflammatory cytokines (IFN-γ and TNF-α; *P* < 0.01), and MDA content (*P* < 0.001). Furthermore, a significant positive correlation was found between these probiotics and liver antioxidant enzymes (SOD, CAT and GSH-Px; *P* < 0.01) and cecal SCFAs (valeric acid, isobutyric acid, butyric acid and isovaleric acid; *P* < 0.001). However, *Ruminococcus 1, Prevotellaceae NK3B31 Group*, and *Desulfovibrio* were completely different from the probiotics above mentioned, exerting an opposite effect. Additionally, the levels of SCFAs and liver antioxidant enzymes showed negatively correlated with liver injury, while the levels of MDA and inflammatory factors were positively correlated with liver injury. The above results showed the changes in intestinal flora regulated by gastrodin is beneficial to improve liver damage induced by PFOA exposure.

Red indicates positive correlation; blue indicates negative correlation. *P < 0.05, **P < 0.01, ***P < 0.001.

## 4. Discussion

In this study, gastrodin treatment significantly relieved PFOA-induced liver inflammation and antioxidant stress via changing the composition and abundance of intestinal flora. Furthermore, gastrodin treatment significantly increased the level of SCFAs, such as butyric acid and isobutyric acid.

Exposure to PFOA causes excessive production of ROS and inflammatory factors, which results in liver damage [5,7]. The activities of SOD, GSH-Px, and CAT were increased but levels of MDA and inflammatory factors were decreased after the administration of gastrodin in this study, which showed that gastrodin can alleviate the liver injury caused by PFOA.

SCFAs are the main metabolites of dietary fiber metabolized by intestinal symbiotic bacteria, which provide energy for epithelial cells and can be transported to the liver through the portal vein to regulate the energy metabolism, so as to alleviate liver diseases [31]. In this study, the levels of SCFAs, including butyric acid, isobutyric acid, valeric acid, and isovaleric acid were downregulated after PFOA exposure, but the administration of gastrodin reversed the downregulation, which might be related to abundance increase of SCFA-producing bacteria, such as Lachnospiraceae (*Lachnospiraceae NK4A136 group* and *Lactobacillus*), Ruminococcaceae (*Ruminococcaceae UCG-014* and *UCG-005*) and Bifidobacteriaceae (*Bifidobacterium*). Therefore, gastrodin is likely to exert protective effect against PFOA-induced liver injury by the abundance increase of SCFA-producing bacteria.

The exposure of PFOA resulted in significant changes in the composition of intestinal flora, while the disordered flora was reshaped by gastrodin treatment, indicating that gastrodin might protect the liver by affecting the abundance and composition of gut microbiota. The administration of gastrodin increased the F/B ratio, which is a key biomarker of gastrointestinal function and intestinal homeostasis [32]. Additionally, at the genus level, gastrodin significantly restored the abundance of symbiotic probiotics, such as *Lactobacillus* and *Bifidobacterium*, which play a significant role in regulating intestinal flora balance, reducing oxidative stress and inflammation, and improving liver pathological changes [33]. Thus, gastrodin may exert protective effects against liver damage through improving gut microbiota dysbiosis. But We have to admit that specific bacteria transplantation *in vivo* will be helpful to explain our conclusion in this study.

## 5. Conclusion

Gastrodin can effectively alleviate liver injury caused by PFOA exposure through improving the intestinal flora dysbiosis. This study provides novel insights into protective liver drugs exploration.

## Supplementary Material

Supplemental Material

## Highlights

PFOA-induced liver injury is correlated with gut microbiota dysbiosis.

Gastrodin can alleviate PFOA-induced liver injury.

Gastrodin exerts hepatoprotective effect via modulating gut microbiota.
