# Supplementary figures and images for: Gastrodin attenuates perfluorooctanoic acid-induced liver injury by regulating gut microbiota composition in mice
Source: Bioengineered. 2021 Dec 4;12(2):11546–56. doi: 10.1080/21655979.2021.2009966 (PMC8810172; doi:10.1080/21655979.2021.2009966)

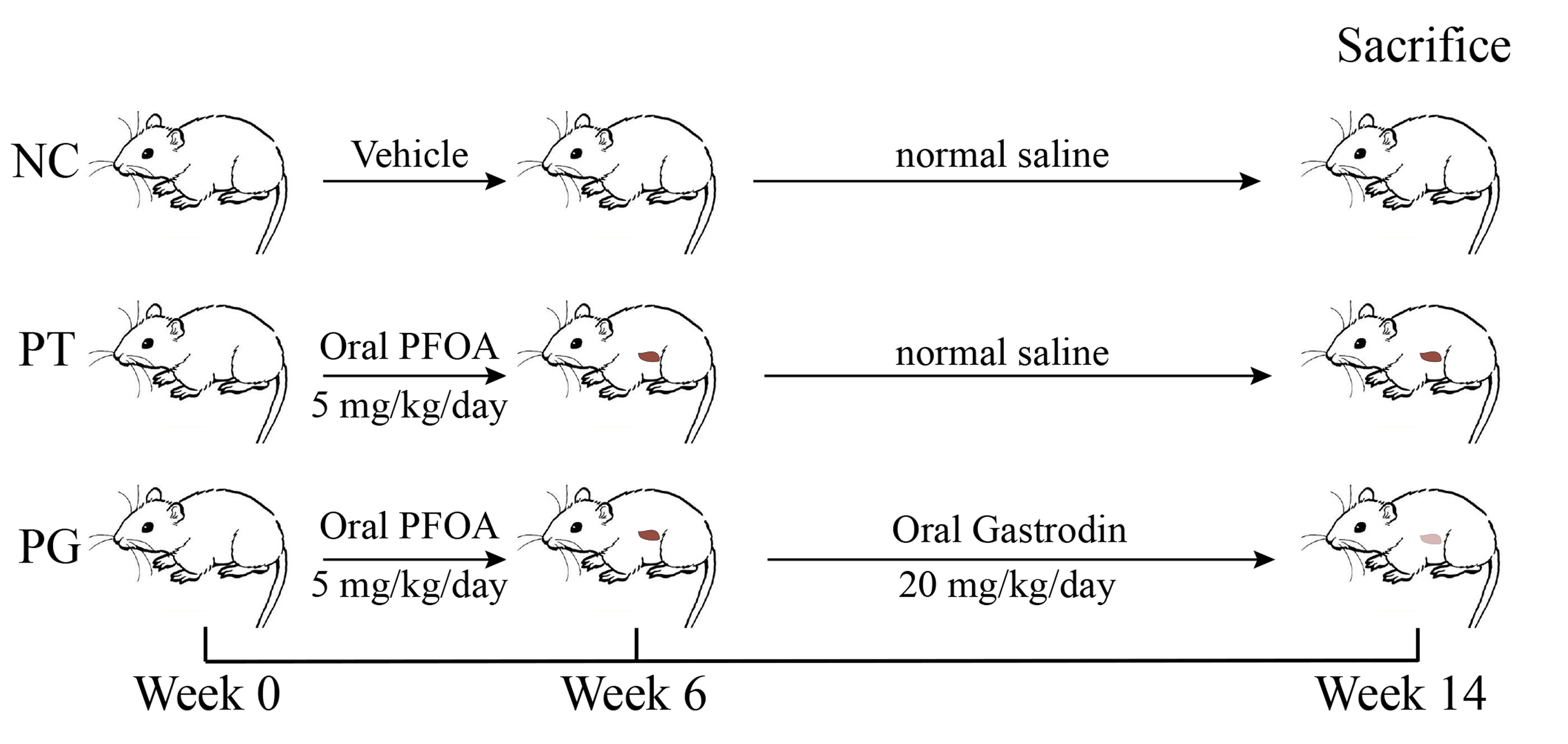

Supplement: Supplemental Material [file KBIE_A_2009966_SM7665.zip › supplementary/Figure S1.tif]

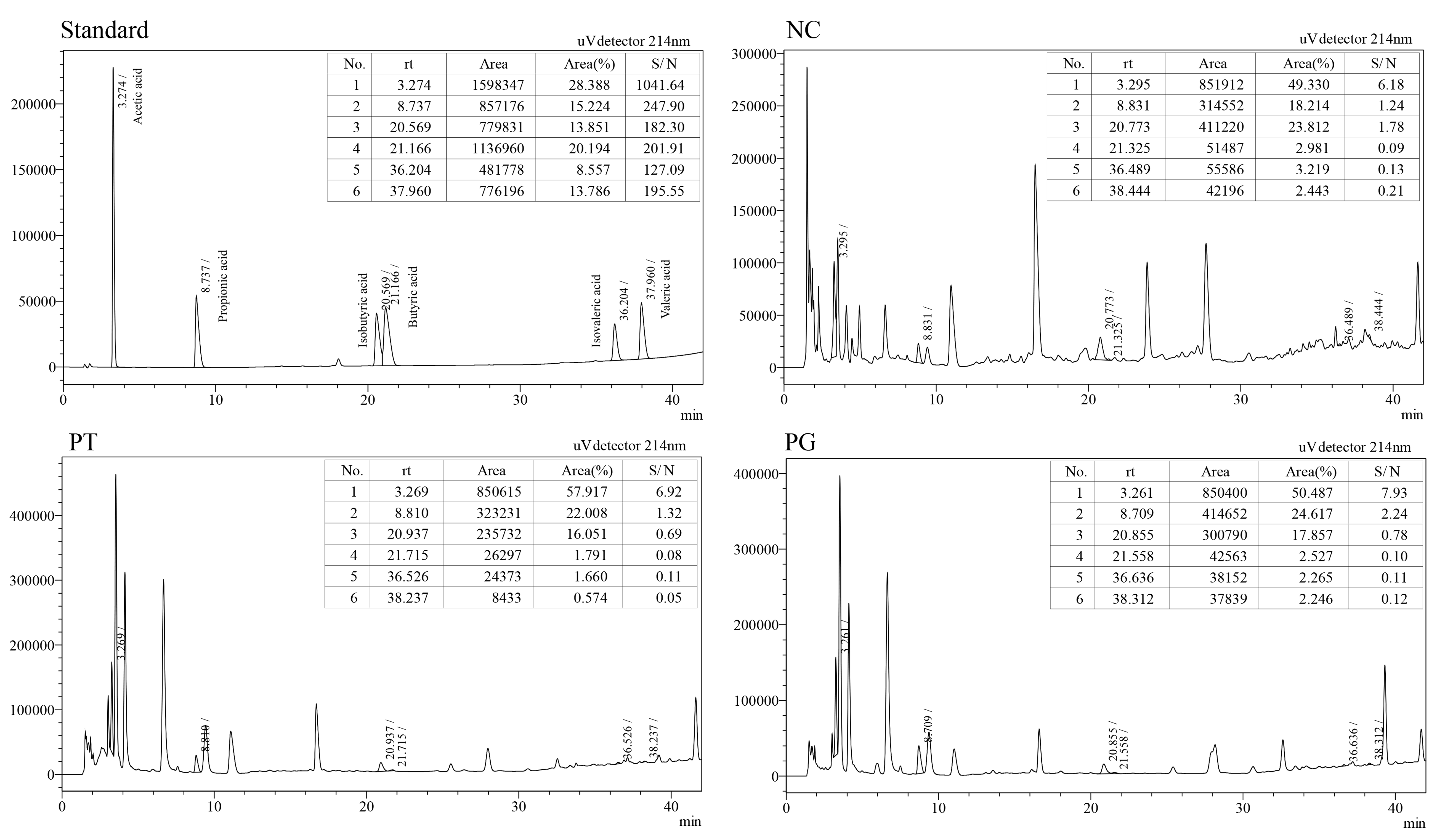

Supplement: Supplemental Material [file KBIE_A_2009966_SM7665.zip › supplementary/Figure S2.tif]
